# The existential crisis of bipolar II disorder

**DOI:** 10.1186/s40345-019-0175-7

**Published:** 2020-01-28

**Authors:** Michael Gitlin, Gin S. Malhi

**Affiliations:** 1 Department of Psychiatry, Geffen School of Medicine at UCLA, Los Angeles, USA; 2 CADE Clinic, Department of Psychiatry, Royal North Shore Hospital, St Leonards, USA; 3 Discipline of Psychiatry, Faculty of Medicine and Health, The University of Sydney, Sydney, Australia

**Keywords:** Bipolar I, Bipolar II, Diagnosis, Nomenclature

## Abstract

The issue of categorical vs. dimensional classification of bipolar disorder continues to generate controversy as it has for generations. Despite the evidence that no psychiatric disorder has discrete boundaries separating pathological and nonpathological states, and within a disorder, no clear differences separate subtypes-which would suggest a more dimensional approach-there are valid reasons to continue with our current categorical system, which distinguishes bipolar I from bipolar II disorder. Complicating the issue, a number of interested constituencies, including patients and their families, clinicians, scientists/researchers, and governmental agencies and insurance companies have different interests and needs in this controversy. This paper reviews both the advantages and disadvantages of continuing the bipolar I/bipolar II split vs. redefining bipolar disorder as one unified diagnosis. Even with one unified diagnosis, other aspects of psychopathology can be used to further describe and classify the disorder. These include both predominant polarity and categorizing symptoms by ACE-activity, cognition and energy. As a field, we must decide whether changing our current classification before we have a defining biology and genetic profile of bipolar disorder is worth the disruption in our current diagnostic system.

## Background

Until 25 years ago, bipolar II disorder was the Pinocchio of psychiatric disorders-long recognized and referred to- but not a “real disorder”. Finally, in 1994, bipolar II disorder was finally given formal recognition in the Diagnostic and Statistical Manual of Mental Disorders (DSM-IV) (American Psychiatric Association 1994). This recognition has continued in all subsequent DSMs with notably little modification of its definition. Thus, as we settled into the new millennium it seemed as if bipolar II disorder had sufficiently matured and achieved full diagnostic status and was now on par with other major mood disorders such as major depression and bipolar I disorder. Indeed, a decade ago it was even deemed worthy of an entire book (Parker 2012). At the same time, its prevalence seemed to be increasing with estimates of an expanded definition (i.e., bipolar spectrum) up to 4.5% in one study and, with definitional modification, 10.9% in others (Merikangas et al. 2011; Angst et al. 2003). Surely, its status was assured.

And yet, in the last few years, a debate has arisen as to the validity and utility of bipolar II as a diagnostic category. Some authors have suggested its elimination (hence, the existential crisis) (Malhi et al. 2019a, b) while others support its continued inclusion in our diagnostic systems (Ha et al. 2019; Nierenberg 2019; Ostacher 2017; Post 2018, 2019; Schaffer 2018; Vieta 2019). In a field as imprecise as ours, it is not surprising that these debates occur. As always, there is at least some merit to both sides of the debate. This paper will summarize both sides of this discussion, highlighting the not always congruent needs of clinicians, patients, researchers and others and the role that this distinction plays in the debate.

## History

As reviewed by Shorter (2012), the classification of mood disorders has a long history with a continued evolution over thousands of years. Although much of the effort prior to the last four decades was to conceptualize and elucidate the relationship between excited states and depressive states, there was frequent acknowledgment about the dimensional nature of mood pathology. More recently, formally defining a subset of manic-depressive illness in which the patient never has marked excited states and at worse they are relatively mild, was first declared by the Research Diagnostic Criteria (Spitzer et al. 1978) in which the term bipolar II first appears. Remarkably, it took 16 more years for DSM to agree that the distinction between bipolar I and bipolar II was worthy of official recognition and include it in its 4th revision.

## Core conceptual issue

As many observers have noted, the core issue in evaluating the merits of bipolar II as a distinct disorder is the creation of a categorical system (such as the DSM or ICD) for what are clearly dimensional forms of psychopathology. This is a problem for all psychiatric diagnoses to some extent and there is no evidence that *any* psychiatric disorder has discrete boundaries that demarcate the disorder either from other psychiatric disorders or normal nonpathological variations of mood, cognition, personality features and so on. This concept is implicitly acknowledged in the multicluster (A, B and C) approach in DSMs in which there was more overlap between some personality disorders (within clusters) than others (across clusters). Dimensional thinking in psychopathology has also given rise to the more colloquial description of spectrum disorders-depressive spectrum disorders, obsessive–compulsive spectrum disorders and, of course, bipolar spectrum disorders. The awkward ‘fit’ of dimensional pathology and measurement into boundary-driven categories is also true for many, but not all, nonpsychiatric disorders. As examples, the boundaries of hypertension and “mild” diabetes create categories out of dimensional measurements, with the definition of pathology shifting over time similar to what is seen in the diagnostic systems for psychiatric disorders. More generally, still, the same can be said for obesity and anorexia nervosa—both of which are subject to cultural factors.

For bipolar II disorder, the purely dimensional approach would be to conceptualize bipolar disorder as one diagnostic entity with variation in the expression of the disorder across individuals (Malhi et al. 2016). In contrast, the categorical approach, currently expressed in our major diagnostic systems, is predicated on the assumption that although there are sufficient similarities to warrant grouping of the disorders there are also sufficient and important distinctions between bipolar disorders (bipolar I vs. bipolar II) to justify the different names/labels (see below for a more detailed discussion of the merits of each of these approaches). As the most egregious example in psychiatry, providing different diagnostic terms and different diagnostic codes to major depression and dysthymic disorder (as occurs in both the DSM and ICD systems) suggests to clinicians, especially those in training, that these are separate disorders even though the preponderance of the evidence suggests that they are one depressive disorder with differences in course. In this way, our diagnostic systems shape clinicians’ thinking, some times in ways that are clinically simply wrong.

## The use of diagnostic categories and systems

A key question that governs the dimensional/categorical distinction in difficult contexts is: for whom are diagnostic categories composed? In other words, who needs or uses them? It would seem that four groups have a stake in this: (1) patients and their families; (2) clinicians; (3) scientists/clinical researchers; and (4) governmental agencies, insurance companies and the legal profession—all have differing needs, make different uses of the diagnoses and require different applications from a diagnostic system.

The first group that has an interest in this issue are patients and their families. As Porter (1997) reminded us, among the central roles that physicians have been tasked with for millennia is giving semantic shape—a label, a diagnosis—to patients’ suffering due to illness. For this purpose, describing a disorder in dimensional terms simply does not suffice for most patients and their families. A term, a categorical diagnosis: “You have a disorder called X” is what is specifically asked for and needed. Does it help patients and their families to distinguish between bipolar I and II disorders vs. combining them within the term “bipolar disorder”? The recent controversy over the decision for DSM-5 to eliminate Asperger’s syndrome as a separate disorder from autism and subsume it under autism spectrum disorders is another example of that controversy (see below for more discussion on this point).

The second group that is clearly invested in diagnosis is clinicians. They have a different set of needs from a diagnostic system as compared to patients and their families. For clinicians, a useful diagnostic system would conform (at least somewhat) with the clinical realities that they see (face validity), be simple enough to use without extraordinary training, provide them with a reliable and widely accepted language for communication and perhaps most importantly have clear prognostic and/or treatment implications. Given these multiple and specific needs, it is difficult (although not impossible) for clinicians to adapt to changes in the diagnostic system. Familiarity and expertise with a recognized system of classification means that there is inevitably significant inertia to change.

The third group, scientists/researchers, rely on diagnostic systems for different purposes than the other three groups. Scientists/researchers have the goals of: (1) elucidating how psychopathology expresses itself in different categories, i.e. to have an accurate representation of how nature is “carved at the joints”. This would require a set of biological and genetic studies to distinguish between related disorders based on scientific data, not somewhat arbitrary decisions by committees. (2) Accurate diagnostic classification based on biology would then lead to more targeted treatment algorithms that employ science/pathology based diagnoses. Analogies in medicine abound but maybe the best example is the distinction of different types of cancer even within the same general name, e.g., breast cancer, based on underlying biology-estrogen positive tumors are treated differently than are estrogen-negative cancers.

A fourth group-government agencies and insurance companies-comprise an interested group in only some countries, most glaringly, the United States. Here, the purpose of diagnoses may be to define psychopathological entities that justify treatment that will then be paid for by governmental or commercial insurances. In the United States, new medications are approved by the Food and Drug Adminstration (FDA) for the treatment of one or more specific disorders. If a medication is approved for treatment of bipolar I disorder but not bipolar II disorder, insurance payors may refuse to pay for treatment for the second (in this case, bipolar II) disorder. Clearly, having one more broadly defined category of bipolar disorder would obviate this problem.

## Lumping vs. splitting: advantages and disadvantages

Each approach-combining bipolar I and II into one disorder vs. keeping them separate solves different problems. Table 1 presents the advantages of each of these two approaches.

**Table 1:** Advantages of *merging* bipolar I and II disorder vs. maintaining distinction

Advantages of merging 1. Conforms to clinical dimensional reality. Truer reflection of clinical picture of illness 2. Promotes greater consistency in treatment approaches 3. Encourages more coherence in bipolar spectrum thinking and research—accommodates mixed states and allows for differential clinical expression of bipolar disorder
Advantages of maintaining distinction 1. Consistent with lack of evidence to support change 2. Less disruptive to patients and families 3. Acknowledges differences in clinical characteristics a. Dominance of depression in bipolar II disorder b. Differential susceptibility to switching c. Greater functional impairment in bipolar I disorder due to destructive nature of manic states

### The advantages of a unified diagnosis

**Fig. 1:**
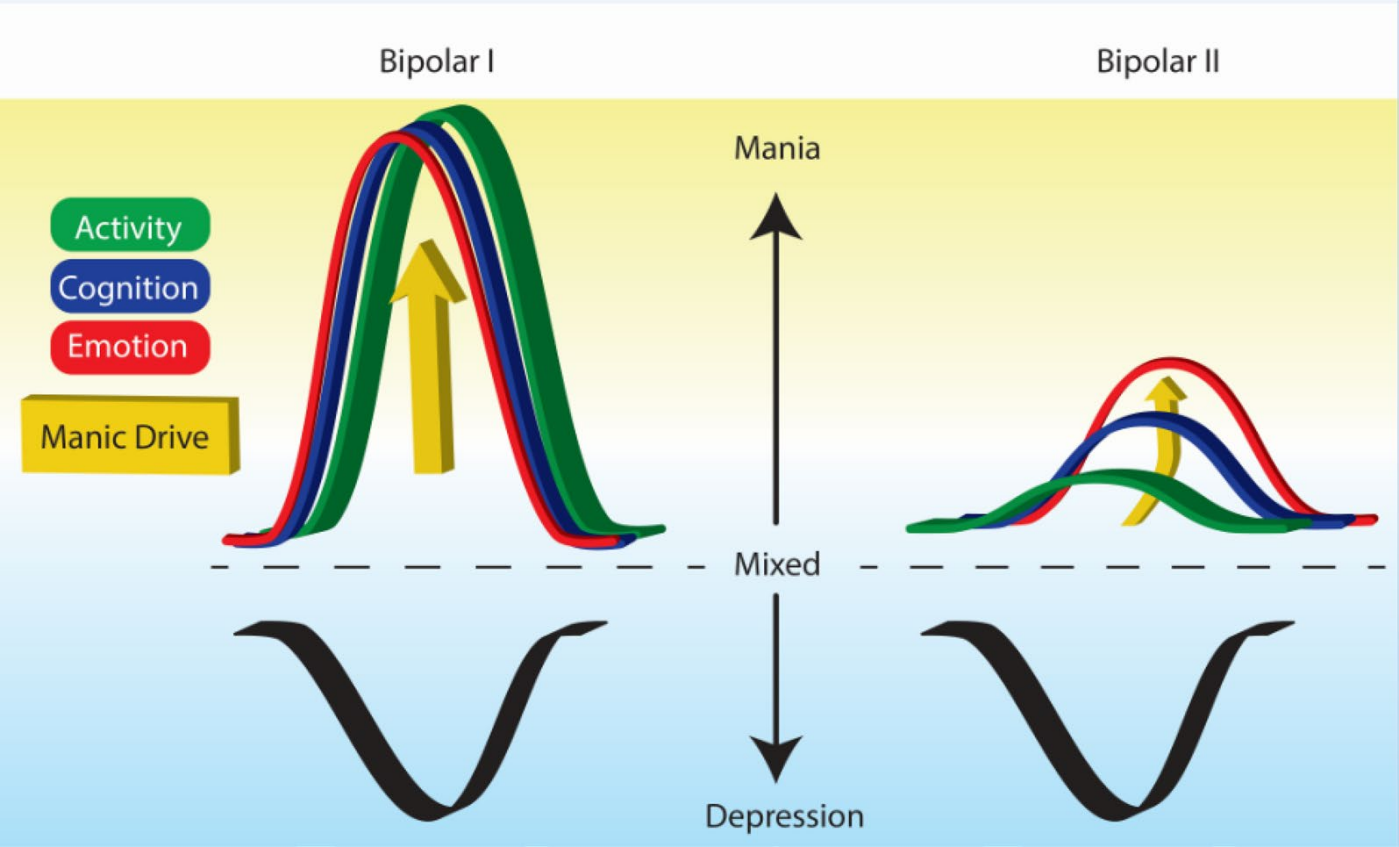
ACE Model of mania. This schematic shows how manic drive, perhaps through differential action on different symptomatic domains can create a seemingly separate phenotype when in fact the difference in manifestation is largely because of the inherent properties of different neurocognitive schema and neural systems within the brain. In florid mania, manic drive (shown in yellow) is so extreme that irrespective of the inherent rigidity of various domains, they are all extended (akin to elastic bands) to the same extent. And so, activity, cognition and emotion are all impacted equally and symptoms from each of these domains are evident. However, when manic drive is more modest, those domains that are inherently more pliant are impacted first and hence why there is separation between emotion, cognition and activity. Emotion, by its very nature is more malleable and variable, whereas cognition succumbs more slowly, and activity is the most hard-wired and therefore requires significant manic drive before it is impacted. The figure also shows that lesser degrees of variation and more subtle changes lead to a more mixed presentation in which features of both mania and depression exist alongside each other, by virtue of belonging to independent domains (ACE). This schematic then explains how ‘bipolar II’ and other putative subtypes could perhaps be created and yet have the same underlying mechanisms and therefore, in essence, remain the same illness

1. Conforms to clinical dimensional reality: The most important and core advantage of a unified single diagnosis is that it is more accurate. It is true—i.e. it reflects the dimensional reality of bipolar disorder. In practical terms it also obviates the need for the non-specific and loosely applied 4-day and 7-day time criteria that are used to define hypomania/mania respectively, and furthermore subsumes the ‘fix’ that DSM-5 has concocted and slipped into its conditions for further study section—namely, ‘short-duration hypomania’—inadvertently acknowledging perhaps the inexactness of its extant definitions. The current formulation of short-duration hypomania in DSM-5 alongside hypomania and mania is confusing because it clearly points to the real-world cut-off between normalcy and manic symptoms as being closer to 2 days as opposed to 4.
  Empirically, it is clear that the intensity and severity of excited states exists on a continuum. One can colloquially describe patients as having “bipolar 1.5” if their excited states are either on the more severe end of hypomania or the milder end of mania, but would it not be both more accurate and simpler to describe this state using the language of severity as one would for any other disorder in medicine-e.g., a milder or a more severe flareup of an autoimmune disorder, for example, or to simply capture the duration by describing how long the symptoms have lasted?
  Combining bipolar I and II disorders (and implicitly, hypomania and mania) also reflects the lack of any biological characteristics that consistently distinguish between these two disorders as currently defined (Vieta and Suppes 2008). Genetically, although some studies have shown that the two subtypes of bipolar breed true; i.e., in the families of bipolar I patients, one sees more bipolar I relatives and only some bipolar II patients, whereas in the families of bipolar II patients, there are a greater number of bipolar II patients with a relative paucity of bipolar Is (Rice et al. 1987 and others), we can simply conceptualize this as reflecting the genetic contribution towards illness severity, similar to the genetic contribution towards cycling frequency (Fisfalen et al. 2005).
2. Conceptually, one unified bipolar disorder would also encourage and allow an overarching set of treatment principles. Of course, the nuances of treating bipolar patients with milder manic states may differ from that in treating more severe bipolar patients. Secondary analyses of studies could then examine potential predictors of treatment response based on severity (e.g., such as the potential risk of a treatment emergent affective switch-TEAS) in order to guide clinicians. Here too, this would be analogous to different treatment approaches of more vs. less severe autoimmune flareups.
3. Having one unified bipolar diagnosis with the inherent understanding of a disorder with dimensional elements would also encourage a more coherent discussion of bipolar spectrum disorders. Currently, with the bipolar I/bipolar II categorical distinction, a mood syndrome characterized by depressive states as well as excited/energized states that do not meet criteria for hypomania are vaguely described as bipolar spectrum disorders. Although a unified bipolar diagnosis would still require some (admittedly arbitrary) boundaries, simply acknowledging the dimensional expressions of bipolar disorder would encourage thinking about spectrum presentations more easily. For example, it would also allow cyclothymia to be included within the bipolar diagnosis, thereby eliminating another rather arbitrary distinction within the current bipolar diagnoses and perhaps limit the likelihood of non-sensical variants such as short-duration cyclothymia (Malhi and Bell 2019). Continuity would also mean that manic number of days could be captured, and other patterns such as mixed states could be included alongside the two extremes of mania and depression.
  One of the key problems in practice is that although the idea of dimensionality is increasingly being accepted, it is still limited mainly to mood, which has maintained primacy when considering mood disorders—perhaps understandably given their name. But in reality, depressive and bipolar disorders, or in traditional terms manic-depressive illnesses, are often an admixture of symptoms that don’t neatly fall into DSM categories of major depression or mania. The current DSM fix for this—namely, mixed features has been justifiably criticized and instead an alternative conception that draws on Weygandt’s and Kraepelin’s concepts regarding mixed states has been proposed. In this model three domains of symptoms have been posited—activity, cognition and emotion (Malhi et al. 2018) with mood subsumed by the latter. Apart from accommodating mixed states as has been described in detail elsewhere (Malhi et al. 2019c), this allows for an appreciation of why bipolar I and II may sometimes appear to be so different (see Fig. 1). Here it is postulated that the pathophysiology that drives mania (*Manic Drive*) differentially affects symptoms within different domains because of their intrinsic properties. Individual symptoms can be regarded as having their own inherent ‘elasticity’ that stems from the nature of the disturbance that causes them, and so when these processes are impacted upon this is reflected in the displacement of the system and creation of various symptoms. The differing properties underpinning each symptom/group of symptoms then leads to the extent to which a symptom is expressed—in other words its severity. In this way a distinction can be seen between Bipolar I in which every system is ‘maxed out’ and stretched to the limit, and bipolar II, in which because some symptoms are more susceptible than others a different pattern of expression is formed and this creates a seemingly different picture—when in fact the constituents are the same (see Fig. 1).

### The advantages of continuing the bipolar I vs. II split

In contrast, there are a different set of reasons for continuing the separation of bipolar I vs. bipolar II disorders some of which provide a different set of advantages.

1. First, why should we merge the two subtypes until we have the biological and genetic data justifying the unity of the two disorders? One day, biological and genetic studies may provide a more scientific basis for distinguishing subtypes of bipolar disorder (or not). That day has not yet arrived. As noted above, the two subtypes breed true in family studies. Should that not be enough to continue the split for now?
2. As noted above, altering diagnostic systems (especially without the biological data not currently available to justify these changes) would burden patients and families. Bipolar II disorder is often seen as a more palatable diagnosis compared to bipolar I disorder. Would bipolar II patients reject the notion of having a bipolar diagnosis because they do not identify with others who have full blown manias?
  Additionally, important clinical characteristics distinguish bipolar I and II disorders. First, depression dominates bipolar II disorder far more than it does bipolar I disorder in both the percentage of time spent in depression and the ratio of depressive to manic/hypomanic times (Judd et al. 2002, 2003). Second, switch rates associated with antidepressants are twice as high with bipolar I vs II disorders (Bond et al. 2008). Consistent with this, when bipolar I patients switch into excited states, they develop full mania 45% of the time and hypomania 55% of the time whereas bipolar II patients who have a TEAS develop a hypomania 95% of the time (Bond et al. 2008). These data suggest that there is a different clinical profile for bipolar II disorder and that it has a different susceptibility to the induction of mania and that as a consequence, optimal treatment algorithms may differ between bipolar I and bipolar II patients. Thus, from a practical perspective keeping the distinction would perhaps help clinicians assess risk/benefit ratios more accurately.
  Furthermore, given the inherently and definitionally greater functional impairment in mania vs. hypomania, distinguishing bipolar I from bipolar II disorders would concretize for clinicians the need to think more carefully about excited states in those labelled bipolar I disorder. This would include both the above mentioned risks with antidepressants [overblown as they might be (Gitlin 2018)], but also the need to more aggressively treat emerging excited/energized states in those with bipolar I disorder, since they are capable of accelerating into a far more destructive state than those with bipolar II disorder.

## Alternative options for classifying bipolar disorder

The proper classification of mood disorders in general, and in particular, bipolar disorder has been a longstanding conundrum. As is currently the case, in the absence of the basic scientific data that would allow a more biologically based classification system, interested observers can only suggest improvements on our current, somewhat arbitrary system. With that caveat, there may be other methods of classifying bipolar disorder.

An example of an alternate method of classifying bipolar disorder utilizes predominant polarity as the central factor, initially described by Angst (1978) and revisited and updated more recently by others (Popovic et al. 2012; Carvalho et al. 2014). Predominant polarity (PP) reflects the relative number and severity of manic vs. depressive episodes within individual patients, defined by at least twice as many episodes of one pole vs. the other (Colom et al. 2006). Patients may be mania predominant, depression predominant or neither, when neither pole dominates the clinical course. Rates of PP differ markedly across different populations but, in general, depressive PP patients outnumber manic PP patients (Pal 2019).

As reviewed elsewhere (Malhi et al. 2019a), other criteria that may also be used to characterize bipolar disorder include duration of episodes and severity of functional impairment. Whether these characteristics can be used to create meaningful diagnostic categories is not clear and perhaps that can be used in the manner that *specifiers* are currently used in DSM-5. The ACE model mentioned previously allows for a transdiagnostic classification of mood disorders—combining depressive and bipolar disorders, including mixed states and allowing for links to anxiety and even psychosis.

## Conceptual and societal consequences of diagnostic categories

Until we have a biological/genetic basis for our mood classification system, our diagnoses will remain tentative at best, based on somewhat arbitrary criteria of severity and/or duration and hence not always clinically useful in terms of prognosticating outcomes or determining optimal therapies. Nonetheless, for now, the question is whether the distinction between bipolar I and bipolar II disorder is clinically useful and should be continued or changed. To address this it is perhaps useful to consider an existing example—the merging of autism and Asperger’s syndrome in DSM-5 into autism spectrum disorder (ASD). This is a critical example of where two disorders have been merged into one broader dimensional category. In that circumstance, the framers of DSM-5 felt that the conceptual unity of autism as a disorder that could be expressed on a continuum outweighed the somewhat arbitrary distinction between Asperger’s as a mild autism spectrum disorder from more classic autism. It is important to note that at times, diagnostic categories become important issues of identity to both patients and their families. This was certainly the case with ASD where there were objections among patients and families about using the term associated with the more severe disorder and patients protesting that “I don’t have autism; I have Asperger’s.” Similarly, perhaps for many patients there is a risk that they would reject the notion of having ‘bipolar disorder’ because their excited so called ‘manic’ states are not as severe as mania and as such they can identify with bipolar II (the milder, and putatively somewhat different form of the illness) but cannot identify with the label ‘bipolar disorder” as it connotes a more severe, more destructive, mania characterized often by psychotic symptoms.

Nonetheless, classification systems do indeed shape as well as reflect clinicians’ and patients’ thinking. Over time, measured in years, not decades, we would all get accustomed to the new diagnostic categories. Reviewing history once again, the emergence of the DSM-III with its new terminology and diagnostic categories is a good example. At first, there were legitimate objections to the somewhat arbitrary nature of the new categories. (e.g., “why would we eliminate anxiety neurosis?” or “What is the relationship of dysthymic disorder to the types of patients we have been seeing in psychoanalytic psychotherapy?”). But gradually the field has accepted these new terms and diagnostic categories have continued to refine the classificatory criteria in subsequent DSMs. Furthermore, the new generation of mental health professionals who grew up with DSM-III and beyond, predictably accepted this system and over time it has become integral to our lexicon and thinking. Thus, perhaps if we merged bipolar I and II into one category with other specifiers (such as predominant polarity), and utilized a model that didn’t assign so much emphasis to mood such as ACE, then after an initial period of adjustment in which there would no doubt be some voicing concerns, objections and even predictions of catastrophic consequences—these would, over time, diminish and give way to gradual acceptance.

## Conclusion

It is our opinion that, from a conceptual viewpoint, eliminating bipolar II disorder would be the most intellectually honest. The question is whether it is worth the work of this change now or whether it would be wiser to wait for the anticipated genetic studies over the coming years and decades until we have a more biologically informed classification system.

## Data Availability

Not applicable.
